# Microfluidic-SANS: flow processing of complex fluids

**DOI:** 10.1038/srep07727

**Published:** 2015-01-12

**Authors:** Carlos G. Lopez, Takaichi Watanabe, Anne Martel, Lionel Porcar, João T. Cabral

**Affiliations:** 1 Department of Chemical Engineering, Imperial College London, London SW7 2AZ, UK; 2 Institute Laue-Langevin, BP 1566 rue Jules Horowitz, 380 42 Cedex 9 Grenoble, France

## Abstract

Understanding and engineering the flow-response of complex and non-Newtonian fluids at a molecular level is a key challenge for their practical utilisation. Here we demonstrate the coupling of microfluidics with small angle neutron scattering (SANS). Microdevices with high neutron transmission (up to 98%), low scattering background (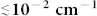), broad solvent compatibility and high pressure tolerance (≈3–15 bar) are rapidly prototyped via frontal photo polymerisation. Scattering from single microchannels of widths down to 60 *μ*m, with beam footprint of 500 *μ*m diameter, was successfully obtained in the scattering vector range 0.01–0.3 Å^−1^, corresponding to real space dimensions of 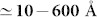. We demonstrate our approach by investigating the molecular re-orientation and alignment underpinning the flow response of two model complex fluids, namely cetyl trimethylammonium chloride/pentanol/D_2_O and sodium lauryl sulfate/octanol/brine lamellar systems. Finally, we assess the applicability and outlook of microfluidic-SANS for high-throughput and flow processing studies, with emphasis of soft matter.

The advent of microfluidics^1^ and rapid prototyping^2^,^3^ provides opportunities to design and generate well-defined flow fields with unprecedented flexibility, ranging from extension to rotation and including pure shear^4^. Despite the general absence of non-linearity associated with inertial forces, fluids confined within microchannels (1–1000 *μ*m), exhibit unexpected and fascinating behaviour^5^. While microflows are generally characterised by low Reynolds numbers, resulting in diffusive and thus slow mixing, simple designs of both continuous and multiphase flows can reduce mixing times to the sub-millisecond range^6^,^7^,^8^ and have enabled major advances in, for example, the study of fast reaction kinetics^6^ or the rapid screening of protein crystallisation^9^. Further, microfluidic approaches are generally high throughput, either sequential or parallel, and require minimal sample consumption, in the pL-*μ*L range^6^,^10^. Complex or non-Newtonian fluids, in particular, can exhibit dramatic microflow response due to the commensurability of characteristic time and length scales^5^. Microfluidic methods can not only precisely quantify their rheological behaviour^11^ but even access hitherto unexplored phenomena that emerge, for example, when large Deborah (De) numbers and low Reynolds (Re) numbers are achieved simultaneously^12^. Soft matter generally responds strongly to flow perturbations exhibiting shear thinning or thickening, thixotropy or rheopexy, but also flow-induced transitions, such as gelation or phase separation^13^,^14^,^15^,^16^,^17^. The understanding of these non-equilibrium phenomena and mapping of ‘flow phase diagrams'^17^,^18^ can therefore be greatly expanded with the plethora of flow types and magnitudes available via rapid prototyping microfluidics.

Small angle scattering is a unique, non-invasive, molecular and nanoscale probe of such structural and kinetic transitions^19^,^20^, and its coupling with microfluidics is thus highly desirable. Complementing an array of *in situ* microscopic, spectroscopic and rheological approaches^5^,^21^,^22^, microfluidic-SAXS^23^,^24^,^25^,^26^,^27^,^28^ has been demonstrated in the high throughput mapping of protein conformation^29^, resolution of sub-ms kinetics of protein folding^23^, nucleation and growth of nanoparticles^30^, or the study of complex fluids under flow, specifically lamellar or particulate solutions under shear and extensional flows^25^,^31^,^32^. While synchrotron SAXS sources offer high brilliance (~10^12^,^13^,^14^ photons s^−1^ at the sample) and small beam sizes (100 *μ*m to sub-*μ*m), scattering contrast in multicomponent soft and biological systems, generally comprising low atomic number species, is fixed and somewhat limited. Small Angle Neutron Scattering (SANS)^19^,^33^ contrast, on the other hand, arises from nuclear interactions which vary markedly for light elements and between isotopes, and significantly between hydrogen and deuterium. Selective deuteration has enabled major advances in soft matter science, from the elucidation of polymer conformation in melts^34^, the reptation mechanism^35^ or exchange kinetics in micelles^36^ to name a few^33^,^37^. Neutrons also offer large material penetration (approximately 90% for 1 cm of aluminium). However, SANS suffers from considerably lower fluxes (~10^8^ neutrons cm^−2^ s^−1^ ) compared to SAXS and limited focussing and collimation, generally requiring longer acquisition times and larger beam sizes, typically 1–1000 s and 10 mm in diameter, respectively. While rheo-SANS is now a well-established technique^38^, flow is generally limited to Couette or cone/plate-plate geometries, stopped flow for rapid mixing and kinetics^39^, or custom made flow cells including Poiseuille^40^, cross-flow^41^, contraction slit^42^ and screw-extruder geometries^43^, with characteristic dimensions in the mm to cm range, which can now be significantly relaxed in microfluidics^44^. However, despite its great potential, the coupling of microfluidics and SANS appears challenging, due to the small scattering volumes associated with microchannel dimensions (≈100 *μ*m) and unfavourable signal-background ratio by common microfluidic matrices (e.g., polydimethyl siloxane elastomers or other hydrogenated polymers, often thermoplastics). In this letter, we tackle this challenge, benefitting from the sustained improvement of neutron sources and advances in rapid-prototyping of microdevices^45^,^46^,^47^,^48^, and investigate the molecular reorganisation of model concentrated surfactant mixtures under flow.

## Results

**Figure 1:**
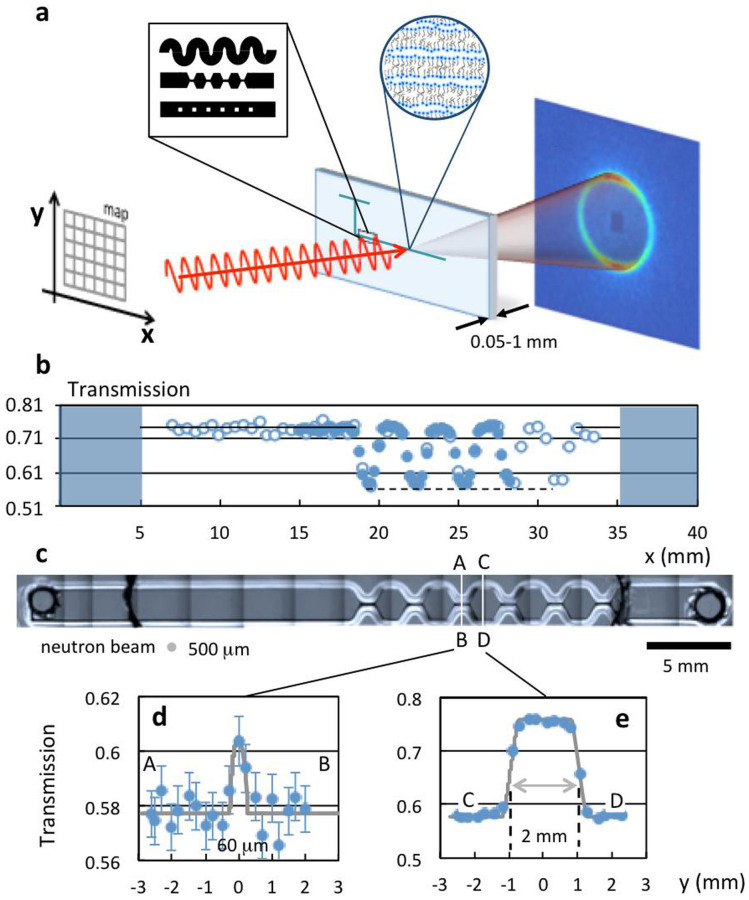
(a) Schematic of a microfluidic-SANS experiment, depicting patterned microchannel geometries and an illustration of a lamellar system, microdevice mounted on a motorised *xyz* stage for spatio-temporal mapping, and 2D scattering pattern. (b) Transmission mapping of the microfluidic device with a 500 *μ*m diameter neutron beam along the microchannel length, with (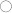) 10 s and (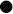) 60 s acquisitions. (c) Optical micrograph of a 6-constriction extensional flow device. (d) Lateral scans (*y* axis) along a wide (C–D) channel and a constriction (A–B). The grey line is the computed normalised transmitted intensity from the beam and microchannel overlap.

Microdevices were fabricated by single-step frontal photo polymerisation (FPP) of a thiol-ene copolymer^45^,^48^ within thin borosilicate or quartz plates, with a mechanically reinforced frame to impart rigidity and enable port connection and operation. Lateral microchannel dimensions from 2 mm to as small as 60 *μ*m are readily achieved, with depths of 100–1000 *μ*m, and a range of flow geometries including periodic contraction-expansion flows, cross-slots and channel height gradients are fabricated. Single channel SANS measurements were demonstrated at the D22 spectrometer at ILL, Grenoble, configured to yield a scattering vector *q*-range of 0.01–0.33 Å^−1^ and typical flux of 5 10^4^ neutron/s at 6 Å incident wavelength (Supplementary Table 1), with typical beam diameter of 500 *μ*m (thus over illuminating the channels). Two concentrated surfactant mixtures were chosen for this study, forming lyotropic lamellar phases with an additional hydrophobic (oil) component, due to their ubiquitous practical application and flow response^49^. Lamellar phases derive their microstructure from a competition between interfacial curvature energy and entropy, and are highly responsive to flow alignment and, in specific flow conditions, hydrodynamic instabilities result in the formation of long-lived metastable phases (e.g. onion phase^17^). We select well-known systems of cetyl trimethylammonium chloride(CTAC)/pentanol/D_2_O^25^ and sodium lauryl sulfate (SDS)/octanol/d-brine^17^,^18^ with characteristic spacings of 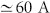 and 150 Å (detailed in Supplementary Information). The experimental setup is illustrated in figure 1(a) and an optical micrograph of a periodic 6-constriction microdevice is shown in (c). Contraction-expansion and crossslot geometries yield primarily extensional flows, which are ubiquitous in the processing of non-Newtonian fluids, ranging from injection moulding, to spraying or spray drying, or continuous flow processing of formulations (including the aforementioned lamellar phases). The SANS experiment starts by generating a spatial microchannel map by fine *xy* neutron transmission scans, illustrated in figure 1(b, d, e), followed by the spatiotemporal mapping at selected positions. Two-dimensional SANS acquisitions as short as 1 s are able to resolve the spatial flow response of these complex fluids at the molecular level.

**Figure 2:**
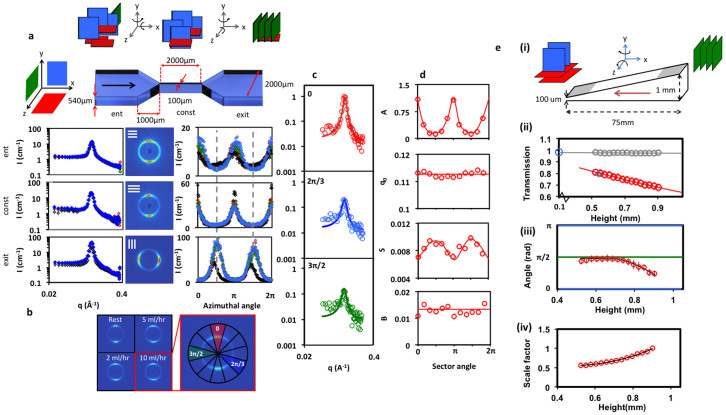
CTAC/pentanol/D_2_O under microflow. (a) Schematic of a microchannel constriction. Green, blue and red sheets represent lamellae orientations in the *yz*, *xy* and *xz* planes as indicated. Radially (left) and azimuthally (right) averaged profiles for positions *E*nt, Const and *E*xit, indicated on the schematic. Black, red, green and blue symbols correspond to, respectively, rest, 2, 5 and 10 mL/hr flow rates. (b) 2D scattering profiles at the constriction as a function of flow rate, and enlarged profile for 10 mL/hr, including azimuthal sectors, analysed in (c) and (d). (c) Representative *I*(*q*) profiles for sectors 0, 2π/3, and 3π/2; lines are fits to a Lorentzian profile *I*(*q*) = *A*/(1+(*q* − *q*_0_)/*S*)^2^ + *B*. d) Fitting parameters A, B, *q*_0_, S as a function of azimuthal angle for 10 mL/hr. e) (i) Schematic of a wedge device, where red arrow indicates flow direction; (ii) transmission of the empty cell (grey 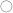, T ≈ 0.98) and sample-filled channel at rest (red 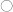); sample transmission follows T = 1.03e^−0.463*h*^ with height *h* [mm], and this trend is is validated for h → 0 and 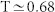 for a sample in 1 mm Hellma cell standard. (iii) Peak angle in the azimuthal average, indicating the main orientation of the lamellae perpendicular to the *xz* plane; the blue lines (0, π) correspond to lamellae oriented in the *xy* plane, and the green line (π/2) corresponds to lamellae oriented in the *yz* plane; (iv) Intensity ratio, referenced to the thickest cross section i.e. (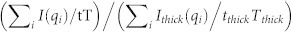) where *t* is the thickness and *T* is the transmission and the subscript thick refers to the 0.9 mm point. The scale factor is thus normalised to 1 at *t* = 0.9 mm.

Figure 2a-d examines the flow response of the CTAC/pentanol/D_2_O lamellar system flowing through a contraction-expansion geometry with flow rates between 0 and 10 mL/hr, corresponding to average velocities of up to 50 mms^−1^. The schematic of the first constriction and selected reference points (entry, constriction and exit) are indicated, along with corresponding 2D scattering profiles, radial and azimuthal averages. As reported earlier^25^, we find that the fluid orients parallel to the flow direction (illustrated by ‘blue' lamellae on the schematic), at the entry and within the constriction, and then perpendicularly (‘green' lamellae) upon exiting the constriction, as it decelerates and experiences an orthogonal extension. This applies to all constrictions in series and at all flow rates examined (including the ‘at rest' condition, corresponding to a fluid introduced at low flow rate (0.5 mL/hr) and allowed to relax for at least 15 min). The radially-averaged scattering profiles are well modelled by a Lorentzian function *I*(*q*) = *A*/(1 + (*q* − *q*_0_)/*S*)^2^ + *B*, where *A* is the height of the peak, *q*_0_ the peak position, *S* the full width half maximum (FWHM) and *B* the incoherent background, in the *q*-range 0.07–0.2 Å^−1^. The integrated scattering intensity and peak sharpness increase as the system flows along the channel. Specifically, the intensity increases by approximately 50% upon entering the constriction, and by an additional 60% upon exiting. As illustrated in Fig. 2a, we interpret these results as arising from a lamellar sheet rotation from the *xz* to *yz* plane, thereby increasing the coherent scattering intensity, as well as from *xy* to the *yz* plane, thus flipping the angle of the structural peak. The radially averaged profiles at the entry and constriction remain unchanged with increasing flow rate. At the exit, however, the scattering intensity nearly doubles compared to the ‘rest' reference and an additional shoulder appears at 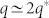, where q* is the position of the main peak, which is not present at other positions and indicates strong lamellar structural alignment normal to the flow direction.

At the constriction entrance, scattering anisotropy increases with increasing flow rate (detailed in Supplementary information Fig S5), as expected for lamellae alignment along the velocity gradient imposed by the constriction. Unexpectedly, however, the azimuthal average corresponding to the system at ‘rest' within the constriction indicates a larger alignment than that under flow, which we associate with the longer residence time experienced. At the exit, the systems under flow show an even greater degree of anisotropy, but now *perpendicularly* to the flow direction, as the lamellae sheets decelerate and rotate upon exiting the constriction (the slight tilt in the ‘rest' pattern is likely due to flow inhomogeneity in these viscous systems). Next we average the 2D scattering profile within the constriction at 10 mL/hr along 12 sectors to obtain *I*(*q*) profiles as a function of azimuthal angle and thus orientation under flow. The angular dependence of the Lorentzian fit parameters is shown in figure 2d. Trivially, the peak height *A* follows the azimuthal profile and we find that its position *q*_0_ remains unchanged, as does the scattering background *B*, within experimental uncertainty. However, along the flow direction, as the peak height *A* increases, its width decreases, quantifying the lamellar alignment upon lateral compression and acceleration within the constriction. At the exit position, at 10 mL/hr, approximately 50% of the scattering intensity lies between ±10° of the flow direction, indicating strong lamellar alignment.

Our FPP approach is also capable of patterning orthogonally to the microchannel direction^45^,^48^ and we next consider a wedge-shaped, or height gradient, microchannel, confined between two quartz windows, depicted in figure 2e (i). Sample injection occurred from the taller towards the thinner channel crossection at constant flow rate 0.5 mL/hr, thus at increasing flow velocity, and the SANS measurements reported were carried out at 10 mL/hr. Figure 2e (ii) shows the transmissions, measured at rest, of the empty and sample-filled channel. The former is found to be constant, at *T* = 0.98, in good agreement with the estimation based on a single quartz plate with a full beam *T* = 0.99^2^ = 0.98, and characteristic of an outstanding neutron cell. The sample transmission in the microchannel decreases exponentially with height *h*, as expected, and validated for *h* → 0 and independent control measurements, allowing precise data calibration and computation of the 3D geometry. Figure 2e (iii) plots the peak angle in the azimuthal average corresponding to the main orientation of the lamellae contributing to coherent scattering (i.e. parallel to the neutron beam). A change from orthogonal alignment with respect to the flow direction at the channel entrance, towards parallel alignment at the thinner section is observed. The scattering intensity, after appropriate thickness normalisation, is found to decrease towards the thinner section of the microchannel wedge, along the velocity and extension gradient, quantifying the relative orientational populations. The intensity ratio, referenced with respect to the thick cross-section, is shown in figure 2e (iv). This decrease accompanies the flow alignment of the lamellae sheets along the velocity direction (and thus perpendicularly to the neutron beam, indicated by ‘green' to ‘red' lamellae in the schematic), in agreement with the flow alignment parallel to the narrower channel walls found above.

**Figure 3:**
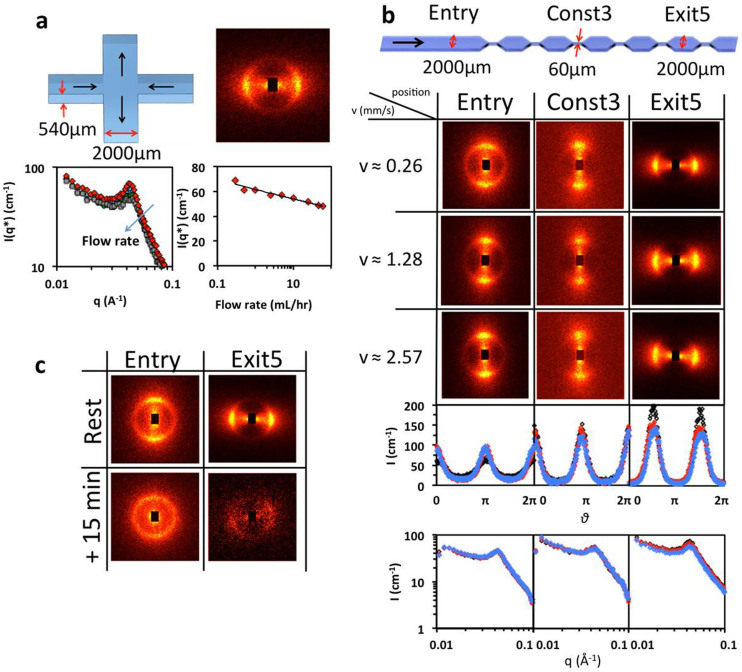
SDS/octanol/d-brine system under microflow. (a) Cross slot device schematic and 2D scattering profile for total flow rate of 0.3 mL/hr; bottom left shows representative radial averages as a function of flow rate and bottom right plots peak intensity *I*(q*) as a function of flow rate. (b) Contraction-expansion series schematic and 2D scattering patterns at flow rates 1 (black), 5 (red), 10 (blue symbols) mL/hr; the mean velocities in the wide channel section (*v*) are shown in the left column with corresponding azimuthal and radial averages. (c) 2D scattering profiles at *Entry* and *Exit5* after cessation of flow, following 0.5 mL/hr, for Δ*t* = 15 and 30 min.

We next investigate an SDS/octanol/d-brine mixture, to evaluate whether similar lamellar rotations, as observed in the CTAC system, are present in multilamellar vesicle (MLV) forming systems^17^,^18^. The characteristic inter-lamellar spacing of MLVs is now ≈150 Å, scattering is generally isotropic, and we re-configure the spectrometer to a lower *q* range (and lower neutron flux). We first report on a cross slot geometry, depicted in figure 3a, generating an extensional flow around the central stagnation point. A representative 2D scattering profile at flow rate 0.3 mL/hr is shown and radially-averaged scattering and peak intensities as a function of flow rates (0.3–60 mL/hr) are computed. The decrease in scattering intensity with flow quantifies the disruption of lamellar ordering by flow, but the insensitivity of the peak position q* with flow rate, within experimental uncertainty, indicates that the lamellar spacing remains unchanged.

The SDS system's response through a series of constrictions is evaluated in figure 3b, showing 2D scattering patterns (positions marked in the schematic) at flow rates of 1, 5 and 10 mL/hr and average flow velocities indicated. The system is aligned from the outset, following injection into the device. At the entry position, the system is least aligned at the lowest flow rate of 1 mL/hr. By contrast, upon expansion (Exit5), the least alignment is found at 10 mL/hr, indicating a correlation between alignment and *residence time* within the device, rather than flow magnitude. An orientational flip at the constriction exit is observed, as found for the CTAC system. The peak positions q* do not vary with flow rate, like in the cross slot geometry, indicating no change of lamellar spacing in this range of flow type and residence time. The overall intensities at 1 mL/hr increase by a factor of 1.5 from the entry position to the constriction and by a further 40% from the constriction to the fifth expansion. At the entry and constriction, the intensities are not significantly affected by flow rate, while at the expansion they decrease by 30% when the flow rate is increased from 1 to 10 mL/hr. Figure 3c considers the structural relaxation of the system over time following cessation of flow after steady state 0.5 mL/hr conditions. Two time points are shown, Δ*t* = 15 and 30 min, enabling the quantification of the relaxation processes both in terms of lamellar orientation and structure.

We have thus resolved the lamellar rotations and alignment experienced by two distinct, representative lamellae-forming systems under different flow types and magnitudes. For the case of contraction-expansion flows, we find that both systems align parallel to the flow before and within the constriction and perpendicularly upon exit, which appear to be general phenomena for anisotropic particle suspensions^31^,^32^. We also find that significant rotation of lamellae from the plane perpendicular to the neutron beam to the planes parallel to the neutron beam occurs both from before to within the extensional fields (induced by constrictions) and further upon exit. Thus, both types of rotations appear to be universal to systems with significantly different molecular structures. From a measurement perspective, we demonstrate 2D SANS data acquisition times down to 1 s, while longer acquisition times (≈5 min from 100 *μ*m channels) yield sufficient statistics for quantitative azimuthal sector analysis.

## Discussion

In this work, we have demonstrated the coupling of microfluidics and SANS, scattering from single microchannels 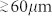 wide. Our microdevices are fabricated using a fast and inexpensive rapid prototyping FPP method, which permits both lateral and orthogonal patterning, as required for precise flow field control. Device fabrication takes less than 1 hr and does not require clean-room facilities or complex equipment.

**Figure 4:**
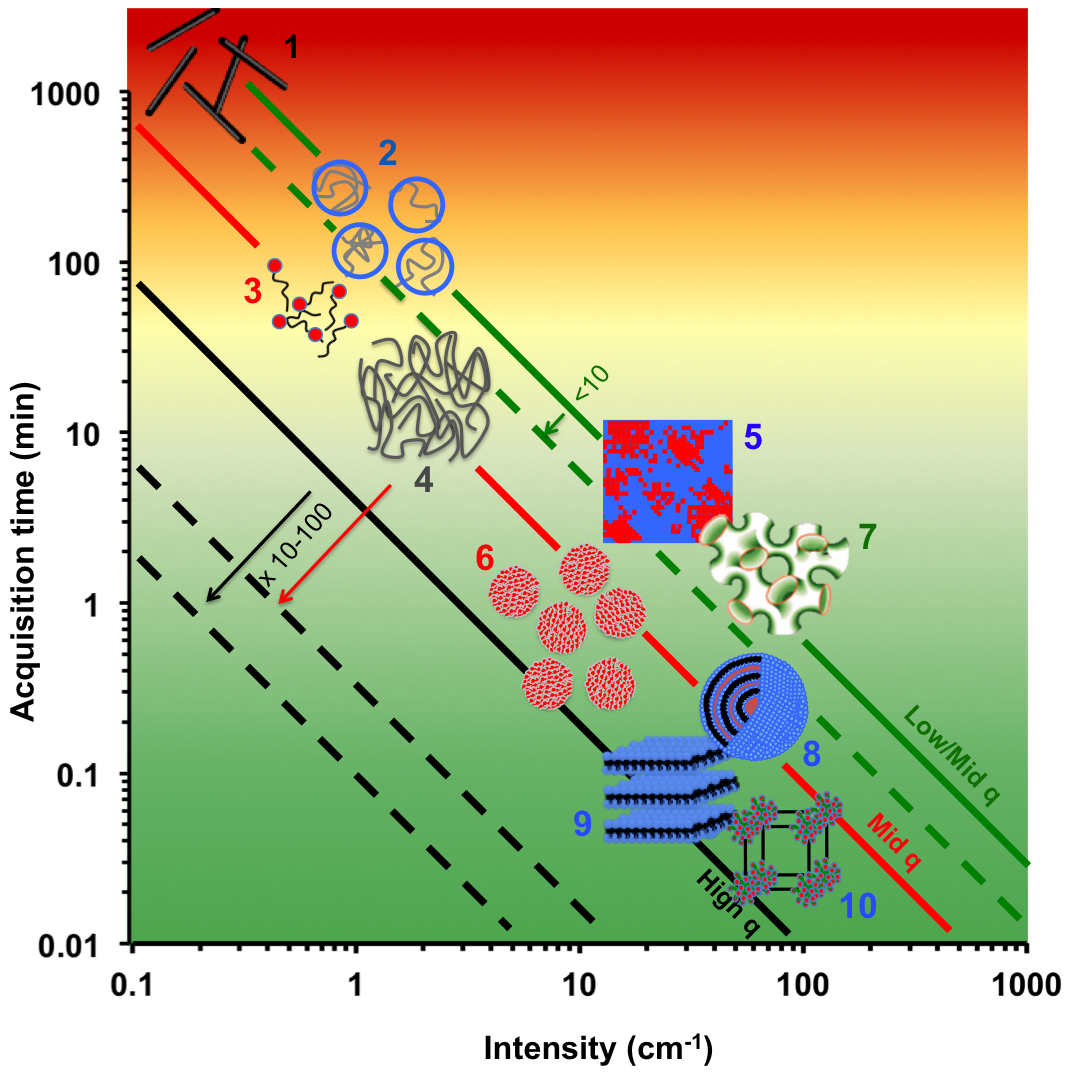
Microfluidic-SANS roadmap, illustrating acquisition times for representative soft matter systems, characterised by typical absolute intensity (cm^−1^), *q*-range and configuration required, referenced to a volume of 0.1 *μl*. Solid lines correspond to representative SANS configu-rations and fluxes for different q ranges: high q (0.05–0.5 Å^−1^), mid q (0.01–0.2 Å^−1^), mid/low q (0.005–0.05 Å^−1^); dashed lines indicate the expected neutron flux increases over the next decade. 1: Dilute carbon nanotube dispersion, 2: Dilute polymer solution, 3: Dilute surfactant solution, 4: Semidilute/concentrated polymer solution, 5: Polymer solution or simple liquid mixture near phase boundary, 6: Concentrated surfactant solution, 7: *L*_3_ phase microemulsion, 8: Multilamellar vesicle emulsion (e.g., SDS system studied in this paper), 9: *L_α_* phase (e.g. the CTAC system studied in this paper), 10: Cubic surfactant phase. We colour-code the background from readily feasible (green) to currently unfeasible microfluidic-SANS experiments (red).

We acknowledge that concentrated surfactant systems form highly scattering, ordered liquid crystalline phases, and that the general suitability of our approach must be further investigated. Based on current SANS instrumentation performance and typical scattering behaviour of model systems, we next establish a ‘capability map' for microfluidic-SANS. We illustrate in Fig. 4 the scattering features of representative soft matter systems, namely the relevant scattering vector ranges, characteristic scattering intensities (in absolute units cm^−1^), and typical corresponding neutron flux. We focus on the interplay between experimental parameters from a user perspective, and estimate the required acquisition times for a reference microfluidic channel of 500 *μ*m depth and a 500 *μ*m diameter neutron beam, viz. a volume of 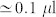 (acquisition times for 100 *μ*m × 100 *μ*m channels would simply read ×25). We populate the graph with systems ranging from weakly scattering dilute polymer solutions, which also require relatively low *q* measurements, to strongly-scattering microemulsion and crystalline systems, generally measured at higher *q*, for which neutron flux is highest. We consider systems requiring less than 1 min acquisition times, to readily enable fine mapping with sub-mm beam sizes, as well as high-throughput experiments, and are thus shown in ‘green'. Other systems, while *compatible* with current SANS capabilities, are limited in the number of acquisitions possible during a standard SANS experiment, which have a typical duration 24–72 hr. Systems whose required acquisition times are greater than 100 min are considered *unfeasible* at present (indicated ‘red' in the graph), and higher flux neutron sources will be required to explore these with microfluidic-SANS. For reference, the mounting and mapping of devices in this work was typically achieved within one hour, and measurement times per state point ranged from a few seconds to a few minutes. The time required to acquire all data presented in this paper, including setup, device mounting, mapping, and equilibration times, was approximately 24 hr, thereby establishing the feasibility of microfluidic-SANS experiments within a typical beam time allocation.

Continued neutron flux improvements will enlarge the range of systems amenable to microfluidic-SANS studies within feasible timescales, indicated by the dashed lines in Fig. 4. The prospect of third generation sources (including the ESS in Sweden^50^) and high performance time-of-flight SANS spectrometers will contribute to further expand the feasibility of microfluidic-SANS. Predicted gains of order 10–100 in flux and wider scattering vector ranges (*q_max_*/*q_min_*) of 3–4 orders of magnitude will allow the interrogation of increasingly small sample volumes and lower sample contrasts. Smaller beam diameters, down to 10 *μ*m, as well as non circular geometries may thus be employed for finer spatial mapping. Throughout this experiment only one (of 3 possible) scattering planes was investigated; through multilevel patterning and device rocking, all flow directions may be accessible. Further to rheological studies of flow-responsive systems, the high-throughput screening of mixtures, in continuous and droplet flows, is expected to become a reality within the near future.

## Methods

### Microdevice fabrication

The microfluidic devices were fabricated following a modified frontal photo polymerisation (FPP) approach^45^,^48^. In short, a thiol-ene copolymer is UV-A polymerised between optically transparent surfaces, through a photomask defining the lateral geometry, and unpolymerised regions are developed by a selective solvent, resulting in the microchannels. In order to minimise scattering background and increase transmission, thin borosilicate or quartz cover slides are employed, with thickness of 140 and 500 *μ*m respectively. Fluid inlets and outlets are drilled through the glass surfaces and capped with connectors for tubing connected to syringe pumps. Thin (~100 *μ*m) microchips are excessively brittle and prone to failure during port drilling and device operation and are therefore mechanically reinforced with a 1 mm thick glass slide with a pre-drilled window around the active microchannels. For this paper, we fabricate passive flow geometries including a rectangular channel with a series of six constrictions, a cross-slot, and a tapered channel, achieving lateral dimensions as small as 60 *μ*m, and effectively with no upper limit (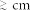).

### SANS setup

The SANS experiments were carried out at the D22 spectrometer of the Institut Laue Langevin, Grenoble, France, with an incident wavelength *λ* = 6 Å, sample-detector distances (SDD) of 2 m and 5.6 m, collimation 2.8 and 5.6 m, yielding a scattering vector range *q* of 0.025–0.33 and 0.01–0.13 Å^−1^ respectively, where elastic scattering vector 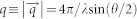, and *θ* is the scattering angle (further detailed in Supplementary Information). Circular cadmium diaphragms restricted the beam diameter to 500 or 1000 *μ*m. The results were normalised and calibrated according to standard procedures (GRASP v6.89). The microdevices were mounted on an upright motorised pedestal with xyz motion and Braintree BS-8000 and Harvard PHD2000 syringe pumps were controlled via LabVIEW.

### Neutron mapping of microdevices

The experimental setup is illustrated in figure 1(a) and an optical micrograph of a 6-constriction microdevice is shown in (c), with depth d = 540 *μ*m, width of wide channel W = 2000 *μ*m, and constriction widths 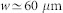. Microchannel mapping is achieved in two stages: a coarse grid map is first achieved by a red laser alignment along the main features, followed by neutron transmission scans of the empty chips along the x and y directions. Representative vertical (y) scans, across wide and narrow channels, are shown in figure 1d; an x-axis scan along the microchannel centreline is shown in figure 1b. The hydrogenous thiol-ene matrix has a 6Å neutron transmission 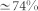, which is sufficient to locate microchannels with 5 s transmission measurements. The microchannel geometry, approximately known from optical microscopy, is directly obtained from precise neutron transmission mapping and subsequently employed in data reduction and calibration.

### Surfactant mixtures

We select two concentrated surfactant mixtures to evaluate the feasibility and potential of microfluidic-SANS. The first is a CTAC/pentanol/D_2_O mixture at ratios 17.4/62.6/20 wt%, which forms an *L_α_* phase with excess water and interlamellar distance 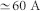
25. Scattering profiles were acquired using a 500 *μ*m diameter beam for 3 min for the positions in the wider channel and for 6 min in the 100 *μ*m constriction. The beam footprint thus over-illuminates the latter and the acquired signal comprises also device background scattering, which is corrected. Such measurements yield good statistics (~10^5^ counts) in the full q range, however 1 s acquisitions suffice to resolve the peak position (detailed in Supplementary information). The second system investigated is an SDS/octanol/d-brine multi-lamellar vesicle (MLV) forming mixture, which has been studied extensively by Roux and co-workers^17^,^18^ by small angle light scattering and microscopy. As the interlamellar peak occurs at lower *q*, we use a 5.6 m SDD to study this system. Scattering profiles were acquired using a 1 mm diameter beam for 5 minutes (corresponding to ~10^5^ counts) for the positions in the main channel and for 10 minutes in the constriction. Data acquisition of 10 s yields reasonable statistics for the full q range and permits azimuthal averaging (detailed in Supplementary information). All samples were injected into the chip at 0.5 mL/hr and allowed to rest for 15 minutes, and are referred to ‘at rest' throughout the paper, although the systems are generally not completely relaxed from the outset.

## Author Contributions

Microdevice fabrication was carried out by T.W. and C.G.L. Spectrometer configuration and optimisation was carried out by L.P. and A.M. C.G.L. performed the SANS data analysis. C.G.L., L.P. and J.T.C. planned and designed the experiment, and prepared the manuscript.

## Supplementary Material

Supplementary Information Supplementary Information
