# Supplementary material for: Microfluidic-SANS: flow processing of complex fluids
Source: Sci Rep. 2015 Jan 12;5:7727. doi: 10.1038/srep07727 (PMC4289890; doi:10.1038/srep07727)
Supplement: Supplementary Information [file srep07727-s1.pdf]

Supplementary Information
Microfluidic-SANS: flow processing of complex fluids

Carlos G. Lopez[a], Takaichi Watanabe[a,b], Anne Martel[c], Lionel Porcar[c] and João T. Cabral[a]

[a]Department of Chemical Engineering, Imperial College London, London SW7 2AZ, UK
[b] Current address: Department of Chemistry and Biotechnology, Okayama University, Japan
[c] Institute Laue-Langevin, BP 1566 rue Jules Horowitz, 380 42 Cedex 9 Grenoble, France

1 Surfactant mixture and formulation

Two representative concentrated surfactant formulations were investigated, based on cetyl trimethylammonium chloride (CTAC) and sodium lauryl sulfate (SDS). CTAC (98.0% (NT)), SDS (ACS reagent, 99.0%), pentanol (puriss. p.a., ACS reagent, 99.0% (GC)), octanol (ACS reagent, 99%) and NaCl (puriss. p.a., 99.5% (AT)) were purchased form Sigma Aldrich and used without further purification. Heavy water (D_2O 99%) was purchased from Sigma-Aldrich. 1g NaCl was added to 50 ml of D_2O to produce d-brine. For both systems, the surfactant was added to the D_2O or d-Brine and shaken until fully dissolved, while heating at 50°C. The oil was added to the solution and manually shaken vigorously. Samples were prepared 72h in advance of measurement to allow stabilisation. In order to ensure reproducibility, three different samples were prepared for each system and measured by SANS in 1mm Hellma cells

with a 12mm diaphragm (full beam) at both spectrometer configurations. The peak position q^*, characteristic of the inter-lamellar spacing, remained unchanged for all three samples. Additional samples, varying the composition for the two systems were prepared by adjusting the oil or aqueous phase content in order to quantify the impact of variations in the formulation on the lamellar structure.

2 Microdevices compatible with SANS

2.1 Microfabrication

Our approach builds upon our frontal photopolymerisation microfabrication method using a commercial multi-functional thiol-ene formulation (Norland NOA-81) as a negative photoresist. Borosilicate microscope slides (1mm thick, Fisherbrand 1238-3118) and coverslips (6631-0146 thickness no. 1, $\simeq 140\ \mu$m), ethanol (Absolute ACS Reagent Ph. Eur.) and acetone (ACS Reagent Ph. Eur.) were purchased from VWR, UK. Quartz slides were purchased from H. Baumbach & Co Ltd. Optical adhesive and photoresist Norland NOA-81 was purchased from Edmund Optics. Nanoports (N333) were purchased from Upchurch Scientific. 1mm glass slides have a neutron transmission of T$\simeq$0.37, and therefore are unsuitable for microfluidic-SANS experiments; we use instead 0.14 mm cover slides, which have a suitable transmission of T$\simeq$ 0.87. However, these are excessively brittle to allow the drilling on inlet/outlet ports and fragile to manual handling. We therefore reinforce the structure with a 1mm glass frame that excludes the microchannel and thus offers high neutron transmission and suitable mechanical integrity. Specifically, a 140 μm (top) slide is UV-glued to a 1mm thick slide with pre-drilled holes in the inlet and outlet positions, and a window for SANS measurement. The photoresist is then sandwiched between the reinforced window and a thin cover slide using 0.54 mm silicon wafers as spacers. A negative photomask is placed on top and the system is exposed to UV-A (365 nm, Spectroline SP100) light at a power of 140 mW/cm^2 for 5 minutes, resulting in a total dose of 42 J/cm^2. The device is placed on a hot plate at 65°C and the channels are flushed with 20 mL of ethanol. A small amount ($\leq$ 1mL) of acetone is then flushed through

the chip immediately followed by injection of 5-10mL of ethanol. This last step is repeated until the uncured photoresist is thoroughly removed and the microchannel is fully developed. Excessive use of acetone leads to degradation of the microchannel features caused by partial swelling. Nanoports were sealed to the inlet and outlet positions using fast curing Araldite (Hunstsman). This procedure was also carried out with 0.5 mm quartz slides instead of thin borosilicate slides, no further modifications are required for this.

2.1.1 Fabrication time and cost estimate

Following the procedure above, the microdevices can be fabricated in approximately 1 h, at relatively low cost and with minimal laboratory requirements. Excluding the Nanoports, tubing and connectors, which are reusable, the above microdevices employing quartz windows cost $\simeq$ £22, while borosilicate chips can be manufactured at a cost of $\simeq$ £2 per device. Access to a clean room is not required and a relatively low power (100 W) mercury flood lamp suffices as a lithography source. These features make this method accessible to a large scientific community interested in flow-SANS studies.

2.2 Microdevice performance

The performance of the devices fabricated as described above is comparable, exhibiting broad chemical resistance (with exception of chlorinated solvents) and able to withstand pressures up to 3-15 bar. The most significant difference between the microdevices is their the neutron transmission: T≈98% for quartz devices and T≈76% for borosilicate devices at typical neutron λ=6Å. In addition, the background scattering from the cell is extremely low in both cases, typically $\lesssim 10^{-2}$ cm^{-1}. Both quartz and borosilicate yield isotropic and approximately flat scattering profiles in the 0.01≤q≤ 0.33 Å^{-1} range, specifically I(quartz) $\simeq$ 0.0025 cm^{-1} and I(borosilicate) $\simeq$ 0.006 cm^{-1}; these are significantly lower than D_2O ($I \simeq 0.05$ cm^{-1}), which is a relatively weak scatterer and ubiquitous sample background. For a microchannel depth of 0.54mm, scattering from our microdevices would be 11 to 14 times lower

(for quartz and borosilicate, respectively) that that of a D_2O filled channel. Given that samples in the green and yellow ('feasible') regions of Fig. 4 of the main paper have scattering intensities $\gtrsim 2$ cm^{-1}, viz. 40 $\times$ larger than I(D_2O), microdevice scattering is negligible compared to sample scattering, which is highly beneficial in minimising data acquisition times and simplifying analysis. For comparison, a conventional Hellma cell scatters approximately 4 or 5$\times$ more than quartz or borosilicate microchips.

3 SANS experimental setup and acquisition

3.1 Microdevice setup

The microdevices were mounted on a custom designed, temperature controlled, upright pedestal frame, such that the neutron beam is normal to the device surface. The pedestal holder is fixed onto an automated xyzθ goniometer breadboard. Syringe pumps, controlled by LabVIEW, drive the flow, and several cameras and a low-power laser beam assist with the microchannel mapping at the start of the experiments. The setup is illustrated in Supplementary Fig. 1.

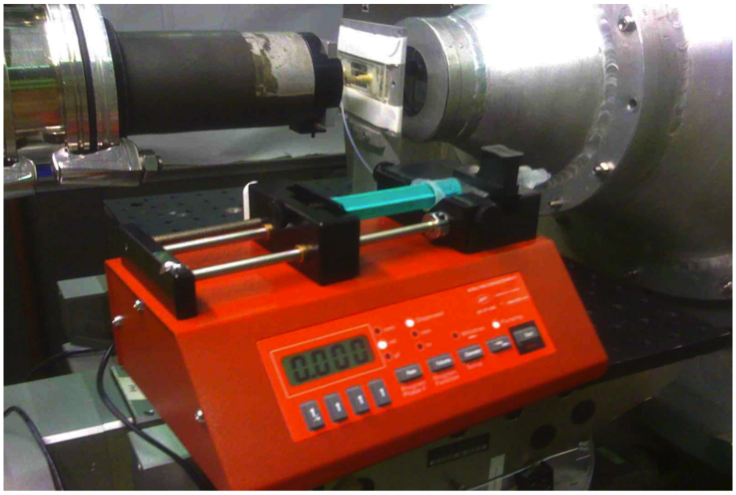

Supplementary Figure S 1: Microfluidic SANS setup, depicting a microchip mounted on the frame/goniometer assembly, a syringe pump, and the neutron incident optics and evacuated detector nose at spectrometer D22, ILL Grenoble.

Supplementary Table 1 shows the wavenumber q-ranges and fluxes obtained for different sample to detector distances (SDD). The flux is reduced by a factor of approximately 275 when reducing the beam diameter from a standard 12 mm ('full beam') to 500 μm by using appropriate cadmium diaphragms.

Supplementary Table S 1: Fluxes for different configurations and beam diameters

SDD	q-range (Å^{-1})	Flux – 12mm beam (neutrons/s)	Flux – 1mm beam (neutrons/s)	Flux – 0.5mm beam (neutrons/s)
SDS1	0.03-0.36	$2*10^7$	$3*10^5$	$7*10^4$
SDS2	0.01-0.13	$7*10^6$		2.510^4

3.2 Acquisition times

An accurate estimate of acquisition times is critical in the design of microfluidic-SANS experiment and to assess its feasibility. Due to the lower fluxes associated

with small beam sizes, acquisition times typically increase by two orders of magnitude. Typical acquisition times employed in this work were 3 min and 5 min for, respectively, the CTAC and SDS systems in the main microchannels, and corresponding to approximately 10^5 total neutron counts. Supplemetary Fig 2a shows the scattering profiles for the SDS formulation, acquired for 5 min (blue) and 10 s (red). These two readings correspond to figure 3c position Exit5 for t=15min (blue) and t=30min (red). The shorter acquisition evidently yields sufficient statistics to resolve the lamellar peak position and a high q power law. Fitting the high q asymptote (q $\geq$ 0.05) with $I = Kq^{-n}$ yields $K = 0.0135 \pm 0.0024$ and n =2.73 $\pm$ 0.07 for the 10s acquisition and $K = 0.0112 \pm 0.0004$ and $n = 2.82 \pm 0.01$ for the 5 min acquisition, within experimental uncertainty.

Suplementary Fig. 2b shows a CTAC system scattering profile for a 3 min acquisition (blue) and for a $\simeq$ 1s acquisition (red). As found above, the peak position and shape can be extracted with sufficient accuracy from the latter. Data outside of the peak region, q $\leq$ 0.07Å^{-1} and q $\geq$ 0.2Å^{-1} is statically noisier, however, and related parameters such as the background cannot be obtained accurately. An azimuthal average around the peak region for the same two data sets are shown in Suplementary Fig. 2d; we conclude that the 2D scattering statistics for 1s acquisition is evidently sufficient to resolve lamellar alignment. As expected, resolving higher order features requires better statistics and longer acquisition times: Suplementary Fig. 2c shows that small shoulder ($\Delta I \simeq 0.3$ cm^{-1}) appearing at q $\simeq$ 0.2Å^{-1} can further be resolved with a 5 min acquisition.

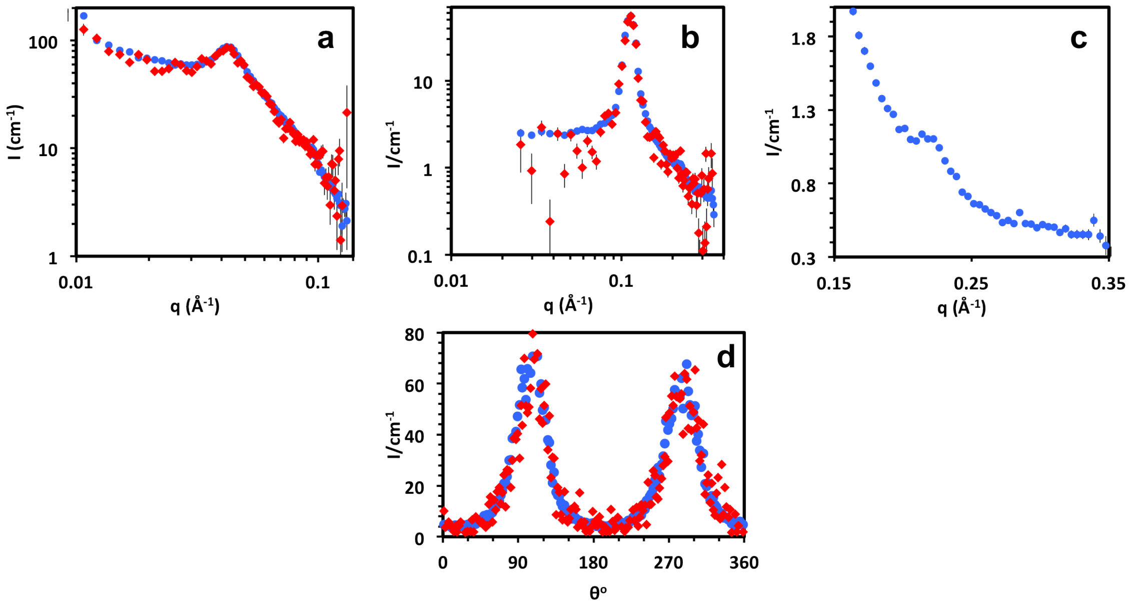

Supplementary Figure S 2: a: Radial averages for SDS system at position Exit 5 as shown in Fig. 3 in the main paper. Red symbols: 10s acquisition, Blue symbols 300s acquisition. b: Radial averages for CTAC system at exit as shown in Fig. 2 in the main paper Red symbols: 1s acquisition, Blue symbols 180s acquisition. c: zoom into high q region of top middle graph (without red symbols), a small feature at $q^{**} \simeq 2q^* = 0.22\text{Å}^{-1}$ can be seen. d: Azimuthal average of the same profiles b.

4 Impact of sample formulation in SANS structure factor

In order to ensure reproducibility and (pseudo)-equilibration of the surfactant mixtures, and assist in the interpretation of results, we have evaluated the dependence of the scattering on the composition of our systems. A ‘full beam’ configuration was employed to decrease SANS measurement time. Suplementary Fig. 3 evaluates the effect of variation in aqueous or oil content and exact sample compositions, along with measured peak position q^*, are given in Supplementary Table 2. Suplementary Fig. 4 summarises the peak positions as a function of the aqueous content for both

systems, indicating that adding water or oil increases the lamellar spacing.

	CTAC (wt%)	D2O (wt%)	Pentanol (wt%)	q* (Å^{-1})
CTAC1	17.40	62.60	20.00	0.112
CTAC2	16.37	64.80	18.82	0.105
CTAC3	15.46	66.76	17.77	0.099
CTAC4	13.25	71.52	15.23	0.086
CTAC5	10.70	77.00	12.30	0.076
CTAC6	17.06	61.37	21.57	0.116
CTAC7	16.42	59.06	24.53	0.112
CTAC8	14.50	52.17	33.33	0.059

	SDS (wt%)	Brine (wt%)	Octanol (wt%)	q*(Å^{-1})
SDS1	6.50	85.60	7.90	0.0425
SDS2	5.99	86.74	7.28	0.0387
SDS3	5.55	87.70	6.75	0.0357
SDS4	4.55	89.92	5.53	0.0268
SDS5	3.50	92.24	4.26	
SDS6	6.45	84.93	8.62	0.0320

Supplementary Table S 2: Sample name, compositions and peak position (q^*) for the different systems studied.

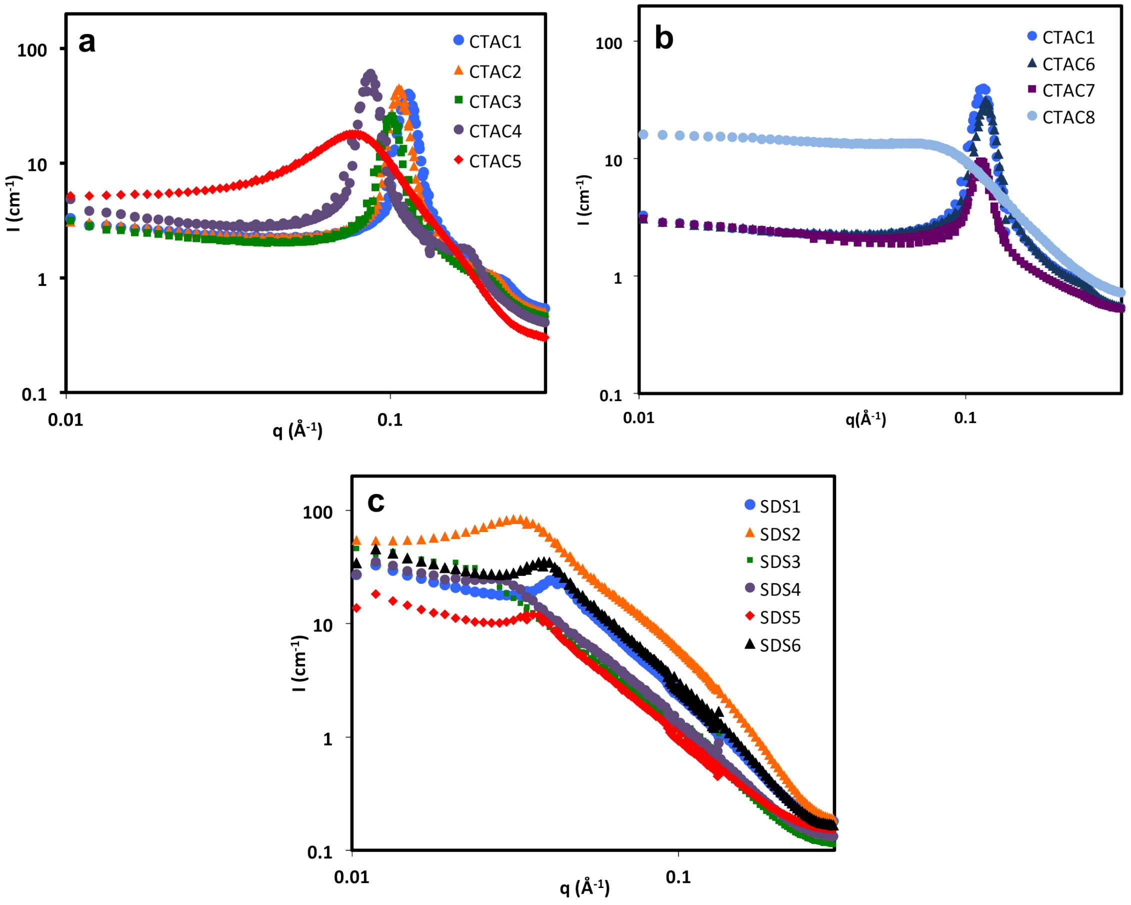

Supplementary Figure S 3: Radially averaged SANS spectra depicting the (a) effect of addition of D_2O to the CTAC reference system; (b) effect of adding pentanol to the CTAC reference system; (c) effect of adding octanol or d-brine to the SDS reference system. Exact mixture compositions are given in supplementary table 2.

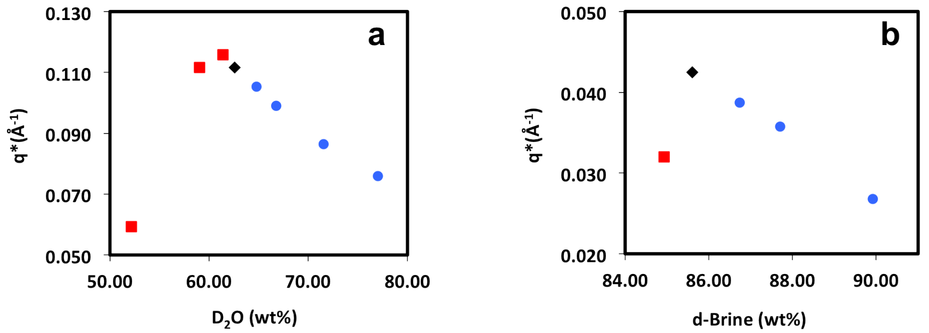

Supplementary Figure S 4: Radially averaged SANS spectra depicting the (a) effect of composition on the lamellar peak position q^* of the CTAC system: (♦) corresponds to sample CTAC1, investigated throughout the paper; (●) corresponds to adding D_2O to the system; (■) corresponds to pentanol addition. (b) Effect of composition on the characteristic q^* SDS system. (♦) corresponds to the reference SDS mixture; (●) corresponds to d-Brine and (■) to octanol addition to the system.

5 Flow alignment at the constriction entrance

Figure 5S shows the azhimutal average for the CTAC system before entering a constriction (Fig. 2a) as a function of flow rate. The anisotropy, quantified as $I_{\theta=\pi}/I_{\theta=\pi/2}$ is seen to increase with flow rate indicating stronger alignment of the lamellae in the direction of flow. The 'rest' case, shows a value of $I_{\theta=\pi}/I_{\theta=\pi/2} \simeq 2$, indicating the system is not fully relaxed.

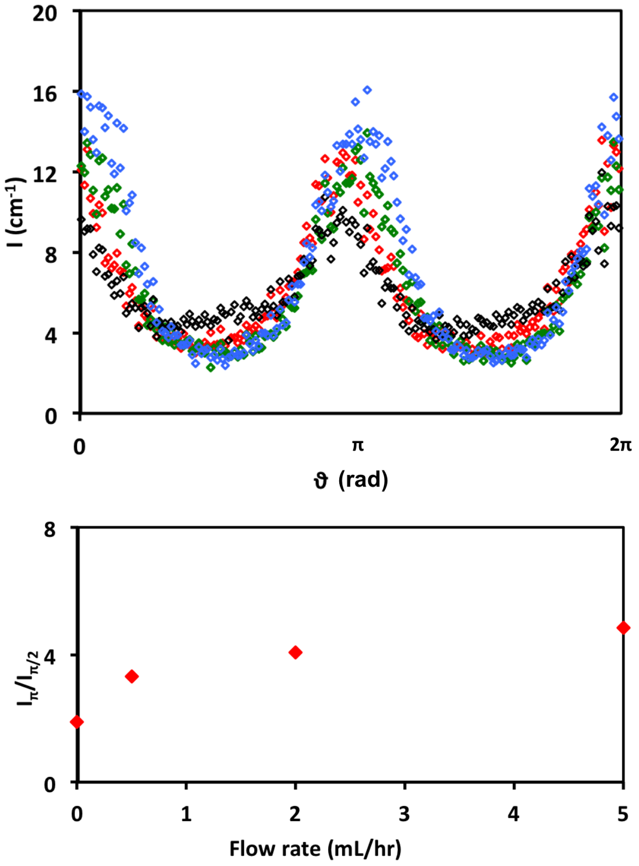

Supplementary Figure S 5: a: Azhimutal average in figure 2a for entry position. Black, red, green and blue symbols correspond to, respectively, rest, 2, 5 and 10 mL/hr flow rates. b: $I_{\theta=\pi}/I_{\theta=\pi/2}$, which serves as a measure of anisotropy is plotted as a function of flow rate.

6 Reorientation within tapered microchannel

Figure S6a shows the schematic of the tapered channel (fig. 2e (i)). The integrated intensity, normalised by thickness (t) and transmission (T) and three representative 2D scattering patterns corresponding to the thick, middle and thin part of the channel for the CTAC system at a total flow rate of 5mL/hr are shown in fig. S6b-c.

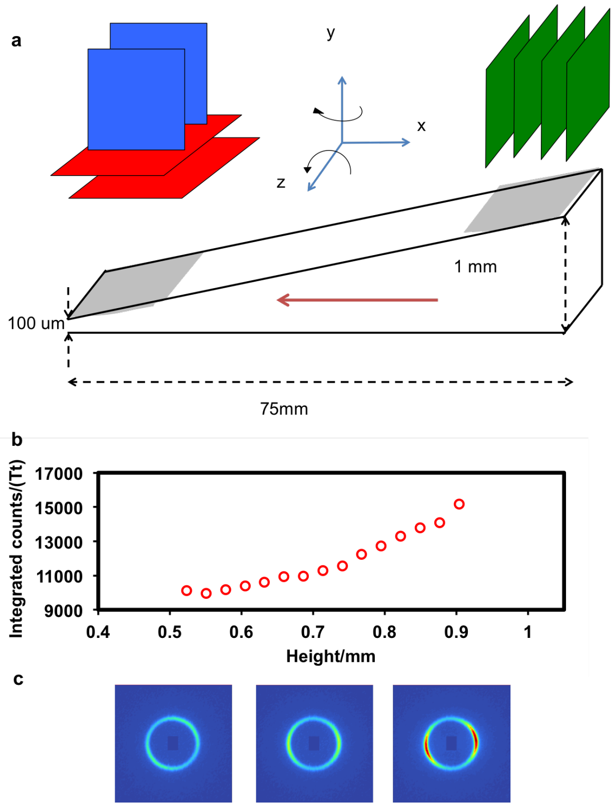

Supplementary Figure S 6: a: Schematic of the tapered channel shown in fig. 2e (i), see the main paper for further details. b: Integrated counts per second, normalised by thickness and transmission. c: 2D scattering profiles corresponding approximately to the position on the chip above.

The normalised integrated intensity is seen to decrease towards the thinner section of the chip indicating lamellae align perpendicular to the neutron beam and hence do not contribute to coherent scattering, i.e. green to red rotations. A further

rotation from green lamellae to blue lamellae can also be seen in the 2D scattering profiles. Towards the thicker part of the channel (i.e. the entry), lamellae are predominantly aligned perpendicular to the flow direction (green lamellae). Further down the channel, a progressive rotation towards alignment parallel to flow (blue lamellae) is observed.
